# Pancreatic cancer masquerading as ischemic enteritis on endoscopy

**DOI:** 10.1002/jgh3.12421

**Published:** 2020-09-30

**Authors:** Enrik John T Aguila, Carlos Paolo D Francisco, Jonard T Co

**Affiliations:** ^1^ Institute of Digestive and Liver Diseases St. Luke's Medical Center Global City Taguig Philippines

**Keywords:** endoscopy, ischemic enteritis, pancreas, pancreatic cancer

## Abstract

Pancreatic tumors usually produce painless jaundice. Other associated symptoms may be secondary, from a direct extension of the tumor, resulting in bowel obstruction. It is extremely rare that pancreatic malignancy presents with ischemic enteritis by invasion of the major arteries, and no report has documented it endoscopically. We present a rare case of pancreatic adenocarcinoma masquerading as ischemic enteritis diagnosed on enteroscopy and endoscopic ultrasound. An initial computed tomography (CT) scan performed in another hospital showed long segmental wall thickening involving the third part of the duodenum to the proximal segment of the jejunum. The patient was referred to our institution for enteroscopy, which showed a poorly distensible third part of the duodenum with purplish mucosa starting at the fourth part of the duodenum until the proximal jejunum. With suspicion of ischemic enteritis, a mesenteric CT angiography was performed, which showed a long segment circumferential wall thickening of the duodenum to jejunum with fullness of the pancreatic head and uncinate process that encases the superior mesenteric artery. Endoscopic ultrasound (EUS) showed a hypoechoic lesion at the head of the pancreas. EUS‐guided fine‐needle biopsy was performed, which revealed pancreatic adenocarcinoma on histopathology.

## Graphical abstract

Despite being a part of the criteria for unresectability of pancreatic malignancies, only very few published reports discuss the sequelae of pancreatic cancer invading the mesenteric vessels. This case report describes a rare case of a metastatic pancreatic disease causing vascular invasion, leading to ischemic enteritis detected endoscopically.

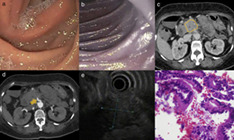

## Introduction

Pancreatic tumors are aggressive tumors with a dismal prognosis. It usually produces painless jaundice, but other associated symptoms may include upper abdominal pain, bloatedness, nausea, vomiting, anorexia, and weight loss. Obstructive symptoms may be secondary from a direct extension of the tumor, resulting in bowel obstruction. It is extremely rare that a pancreatic malignancy presents with ischemic enteritis by invasion of the major arteries. To our knowledge, this is the only case report to describe a metastatic pancreatic disease causing vascular invasion, leading to ischemic enteritis detected endoscopically. Despite being a part of the criteria for unresectability of pancreatic malignancies, only very few published reports discuss the sequelae of pancreatic cancer invading the mesenteric vessels.

## Case report

A 62‐year‐old female with no comorbidities was referred for a 1‐year history of recurrent vomiting associated with bloatedness and weight loss. There was no fever or changes in bowel movement. She had no family history of malignancy. Multiple consults had been carried out but were inconclusive. Her complete blood count, bleeding parameters, and serum electrolytes were unremarkable. A plain abdominal computed tomography (CT) scan performed in another hospital revealed a long segment wall thickening involving the third part of duodenum to the proximal segment of the jejunum. Primary impression was partial bowel obstruction secondary to small bowel thickening due to enteritis of unknown etiology. She was advised to undergo enteroscopy and hence was referred to our institution.

**Figure 1:**
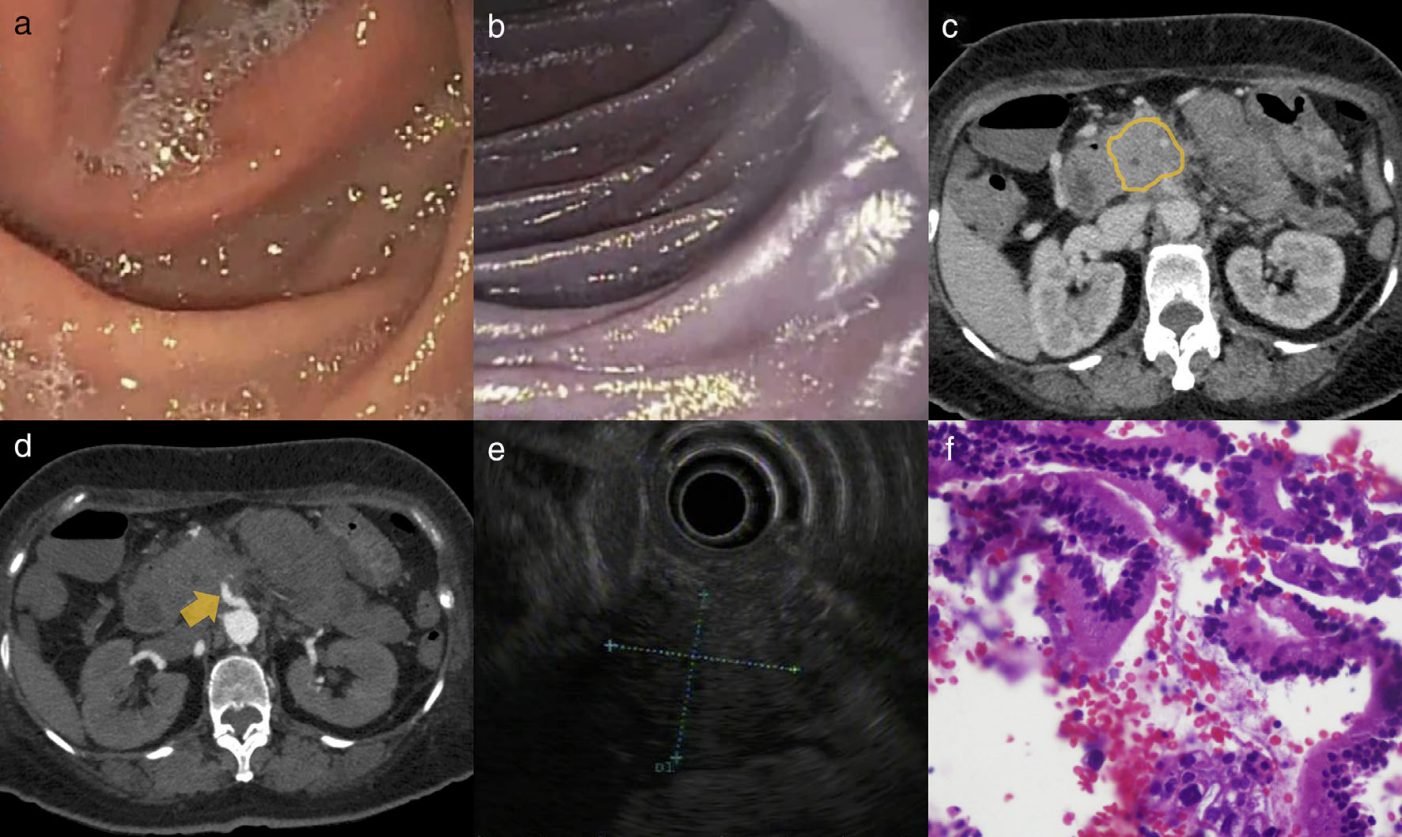
Single‐balloon enteroscopy showed (a) poorly distensible D3 and (b) purplish coloration of the mucosa in D4 until the proximal jejunum. Mesenteric angiography showed (c, yellow line) pancreatic head and uncinate process fullness, causing (d, yellow arrow) encasement and compression of the superior mesenteric artery. Endoscopic ultrasound showed (e, dotted line) a hypoechoic lesion at the head of the pancreas, which encased the superior mesenteric artery and superior mesenteric vein. Histopathology showed (f) findings suggestive of pancreatic adenocarcinoma.

The patient underwent enteroscopy up to the proximal jejunum using a single‐balloon enteroscope (Fig. 1). It showed a normal upper gastrointestinal (GI) tract. The mucosa starting from the D3 was noted to be poorly distensible despite insufflation (Fig. 1a). Starting at D4 (Fig. 1b), the duodenal mucosa was noted to be purplish in color until the proximal jejunum with a length of approximately 15–20 cm. Biopsies were obtained, which showed a focal, mild cryptitis and mildly dilated mucosal capillaries. There was no dysplasia seen. With the biopsy findings, endoscopic diagnosis of ischemic enteritis, and suspicion of mesenteric ischemia, a mesenteric CT angiography was performed, which showed a long segment circumferential wall thickening of the duodenum to the jejunum. There was also fullness seen in the pancreatic head and uncinate process (Fig. 1c) that encases the superior mesenteric artery (SMA) (Fig. 1d). A CA19‐9 was requested, which showed an elevated result at 1209 U/mL (NV: 0.55–1.47).

A diagnostic endoscopic ultrasound (EUS) was performed, which showed a hypoechoic lesion at the head of the pancreas measuring 27.3 × 30.2 mm (Fig. 1e). The lesion was noted to encase the SMA and superior mesenteric vein (SMV). There was no noted vascular thrombus. On elastography, the lesion showed a bluish hue, suggesting that the pancreatic mass has more of a solid consistency. An EUS‐guided fine‐needle biopsy showed atypical columnar epithelium arranged in strips and in cribriform pattern (Fig. 1f). The cells have enlarged, round to ovoid, hyperchromatic nuclei with prominent nucleoli and a scant to moderate amount of mucin‐filled cytoplasm. The final histopathologic diagnosis was pancreatic adenocarcinoma.

## Discussion

Pancreatic cancers can be difficult to diagnose due to their varied manifestations. Tumors of the head of the pancreas produce symptoms earlier in the course of the disease, while tumors located in the tail have silent presentations and are usually diagnosed late in the course after extensive local growth or when metastasis has occurred. Early detection of pancreatic tumors would have better prognosis, hence the need for a high index of suspicion.

The patient was referred to our institution for enteroscopy as a prior CT scan was inconclusive likely because of the lack of contrast. With the reason of referral and the patient's clinical history of persistent nausea and vomiting, performing a single‐balloon enteroscopy would be beneficial in the direct visualization of the affected bowel segment with the differential diagnoses of tumor, infection, or inflammatory bowel disease. It is able to examine the small intestine more extensively into the jejunum and/or ileum. Likewise, histopathologic diagnosis can be obtained, or therapeutic measures can be taken with an enteroscopy. Reported diagnostic yields have ranged from 41 to 65% and therapeutic yields from 7 to 50%.^1^ A study by Wang *et al*. has shown that the diagnostic value of balloon enteroscopy is better than performing a multidetector CT enterography for small bowel diseases.^2^ In our patient, the enteroscopy was able to visualize up to the jejunum where the purplish mucosa was noted, hence the diagnosis of ischemic enteritis.

The clinical relevance and prognostic value of obtaining a biopsy with histological ischemia is unknown. Biopsy is not routinely performed during endoscopy unless endoscopic findings are extensive, atypical, or there is suspicion of malignancy.^3^ In this case, a biopsy was obtained from the purplish duodenal mucosa, which showed findings suggestive of congestion. This correlates with the patient's endoscopic findings suggestive of ischemia. Vascular congestion is one of the cardinal pathohistological features of ischemia aside from edema and coagulative mucosal necrosis.

With the endoscopic finding suggestive of ischemic enteritis, performing a mesenteric CT angiography serves as the next imaging method of choice necessary in the diagnostic algorithm. Mesenteric CT angiography can accurately diagnose intestinal ischemia with a sensitivity of 93.3% and a specificity of 96%.^4^ The patient's mesenteric angiography showed a pancreatic head and uncinate fullness causing encasement and compression of the superior mesenteric vessels. A contrast‐enhanced CT scan may sometimes reveal fullness or indeterminate pancreatic enlargement but may be difficult to distinguish it from a mass.^5^ In this case, tumor invasion of a probable pancreatic malignancy to the major vessels was highly suspected. A serum CA 19–9 was also requested for the patient, which revealed an elevated result at 1209 U/mL (normal value: 0.55–1.47).

Performing an endoscopic ultrasound is considered the most accurate single test for the diagnosis of pancreatic cancer and has higher sensitivity and specificity than the usual CT scans.^6^ An EUS‐guided biopsy can be conducted, and it has been the most widespread technique for tissue acquisition of suspicious pancreatic lesions.^7^ It is, however, limited to endoscopists with expertise as it is highly operator‐dependent. Our patient underwent EUS both to evaluate the pancreatic head fullness and to obtain pancreatic tissue specimens for a more definitive diagnosis.

Surprisingly, despite being a part of the criteria for unresectability of pancreatic malignancies, only two case reports are published discussing the sequelae of pancreatic cancer invading the superior mesenteric vessels. A study by Nitta *et al*. described a 73‐year‐old woman with pancreatic cancer receiving chemotherapy who presented with abdominal swelling and persistent vomiting.^8^ CT scan showed occlusion of the SMA by pancreatic cancer; hence, the patient underwent emergency laparotomy where the large necrotic and ischemic intestine was resected. Another case report by Elsiddig *et al*. described a 50‐year‐old male with acute thrombosis of the celiac trunk due to an advanced pancreatic cancer.^9^ He presented with upper abdominal pain and coffee‐ground vomitus. CT scan showed a big pancreatic mass in the neck. Emergency laparotomy was performed, which revealed the pancreatic mass infiltrating the celiac trunk, with a gangrenous stomach and intestines.

This case report is notable as the patient has been diagnosed with pancreatic cancer with the aid of a battery of tests unlike the published case reports where patients have already been diagnosed previously. In retrospect, we acknowledge that this case could have been diagnosed earlier if a more appropriate imaging method was used in the previous hospital. Nevertheless, this case is unique as the diagnosis was made endoscopically with documentation of the purplish discoloration of the duodenal mucosa, and no published report has described this presentation yet. As such, this case report serves as a vital addition to the current knowledge of pancreatic malignancies and their manifestations.

## References

[1] Marques M , Antunes J , Coelho R *et al* Single‐balloon enteroscopy efficacy and degree of concordance with noninvasive evaluation of small bowel. Endosc. Int. Open. 2017; 5: E96–E102. 28210706 10.1055/s-0042-121415 PMC5305424

[2] Wang J , Guo Q , Zhao J *et al* Multidetector CT enterography versus double‐balloon enteroscopy: comparison of the diagnostic value for patients with suspected small bowel diseases. Gastroenterol. Res. Pract. 2016; 2016: 5172873. 26962305 10.1155/2016/5172873 PMC4707381

[3] Herman J , Chavalitdhamrong D , Jensen DM , Cortina G , Manuyakorn A , Jutabha R . The significance of gastric and duodenal histological ischemia reported on endoscopic biopsy. Endoscopy. 2011; 43: 365–8. 21360426 10.1055/s-0030-1256040 PMC3816952

[4] Menke J . Diagnostic accuracy of multidetector CT in acute mesenteric ischemia: systematic review and meta‐analysis. Radiology. 2010; 256: 93–101. 20574087 10.1148/radiol.10091938

[5] Miller FH , Rini NJ , Keppke AL . MRI of adenocarcinoma of the pancreas. Am. J. Roentgenol. 2006; 187: 365–74. 10.2214/AJR.05.0875 16985107

[6] Bhutani MS , Koduru P , Joshi V *et al* The role of endoscopic ultrasound in pancreatic cancer screening. Endosc. Ultrasound. 2016; 5: 8–16. 26879161 10.4103/2303-9027.175876 PMC4770628

[7] Puli SR , Bechtold ML , Buxbaum JL , Eloubeidi MA . How good is endoscopic ultrasound‐guided fine‐needle aspiration in diagnosing the correct etiology for a solid pancreatic mass? A meta‐analysis and systematic review. Pancreas. 2013; 42: 20–6. 23254913 10.1097/MPA.0b013e3182546e79

[8] Nitta N , Yamamoto Y , Sugiura T *et al* A case of pancreatic cancer invading the superior mesenteric artery causing extensive intestinal necrosis that was successfully treated by surgery. J. Surg. Case Rep. 2019; 4: 1–4. 10.1093/jscr/rjz118 PMC6479187 31044060

[9] Elsiddig HD , Jabra IO , Dabora AA , Ibrahim SZ . An unusual presentation of advanced pancreatic cancer: coeliac axis occlusion and acute upper gut ischemia. Open Access J. Transl. Med. Res. 2017; 1: 114–15.

